# Human embryonic stem cell-derived extracellular vesicles alleviate retinal degeneration by upregulating Oct4 to promote retinal Müller cell retrodifferentiation via HSP90

**DOI:** 10.1186/s13287-020-02034-6

**Published:** 2021-01-07

**Authors:** Yifeng Ke, Xiaoe Fan, Rui Hao, Lijie Dong, Min Xue, Liangzhang Tan, Chunbo Yang, Xiaorong Li, Xinjun Ren

**Affiliations:** 1 Tianjin Key Laboratory of Retinal Functions and Diseases, Tianjin International Joint Research and Development Centre of Ophthalmology and Vision Science, Eye Institute and School of Optometry, Tianjin Medical University Eye Hospital, No 251, Fukang Road, Nankai District, Tianjin, 300384 People’s Republic of China; 2 Jincheng People’s Hospital, Jincheng, 048000 Shanxi People’s Republic of China; 3 Tianjin Eye Hospital, Tianjin Key Laboratory of Ophthalmology and Vision Science, Nankai University Eye Hospital, Clinical College of Ophthalmology, Tianjin Medical University, Tianjin, 300020 People’s Republic of China; 4 Department of Ophthalmology, Anhui No.2 Provincial People’s Hospital, Hefei, 230000 Anhui People’s Republic of China; 5 Institute of Genetic Medicine, Newcastle University, Central Parkway, Newcastle upon Tyne, NE1 3 BZ UK

**Keywords:** Retinal degeneration, Retinal Müller cell, Human embryonic stem extracellular vesicle, Microvesicle, HSP90, Oct4, Retrodifferentiation

## Abstract

**Objective:**

Retinal degenerative diseases remain the dominant causes of blindness worldwide, and cell replacement is viewed as a promising therapeutic direction. However, the resources of seed cells are hard to obtain. To further explore this therapeutic approach, human embryonic stem extracellular vesicles (hESEVs) were extracted from human embryonic stem cells (hESCs) to inspect its effect and the possible mechanism on retinal Müller cells and retinal function.

**Methods:**

hESEVs were extracted by multi-step differential centrifugation, whose morphologies and specific biomarkers (TSG101, CD9, CD63, and CD81) were observed and measured. After hESEVs were injected into the vitreous cavity of RCS rats, the retinal tissues and retinal functions of rats were assessed. The alteration of Müller cells and retinal progenitor cells was also recorded. Microvesicles (MVs) or exosomes (EXOs) were extracted from hESCs transfected with sh-HSP90 or pcDNA3.1-HSP9, and then incubated with Müller cells to measure the uptake of EVs, MVs, or EXOs in Müller cells by immunofluorescence. The retrodifferentiation of Müller cells was determined by measuring Vimentin and CHX10. qRT-PCR and western blot were used to detect HSP90 expression in MVs and evaluate Oct4 level in Müller cells, and Co-IP to inspect the interaction of HSP90 and Oct4.

**Results:**

RCS rats at the postnatal 30 days had increased retinal progenitor cells which were dedifferentiated from Müller cells. hESEVs were successfully extracted from hESCs, evidenced by morphology observation and positive expressions of specific biomarkers (TSG101, CD9, CD63, and CD81). hESEVs promoted Müller cells dedifferentiated and retrodifferentiated into retinal progenitor cells evidenced by the existence of a large amount of CHX10-positive cells in the retinal inner layer of RCS rats in response to hESEV injection. The promotive role of hESEVs was exerted by MVs demonstrated by elevated fluorescence intensity of CHX10 and suppressed Vimentin fluorescence intensity in MVs rather than in EXOs. HSP90 in MVs inhibited the retrodifferentiation of Müller cells and suppressed the expression level of Oct4 in Müller cells. Co-IP revealed that HSP90 can target Oct4 in Müller cells.

**Conclusion:**

hESEVs could promote the retrodifferentiation of Müller cells into retinal progenitor cells by regulating the expression of Oct4 in Müller cells by HSP90 mediation in MVs.

## Introduction

Retinal degeneration (RD), including retinitis pigmentosa (RP) as well as age-related macular degeneration, remains the primary causation of irreversible blindness in the developed countries with the pathology of the light-sensing photoreceptors missing [1, 2]. Photoreceptors are immensely specialized sensory neurons that widely presented in a variety of organisms to capture light and initiate vision [3]. The operation of the mammalian visual system is performed by switching between rod and cone photoreceptors [4]. As the loss of rod and secondary death of cones, visual acuity is declined and eventually leads to blindness [5]. In recent years, much attention has been attached to the identification of therapeutic approaches for retinal regeneration. Several therapeutic approaches for retinal degenerative diseases are currently being developed [6, 7]. However, the regulatory mechanism of retinal regeneration remains largely unexplored.

Retinal pigment epithelium (RPE) cells and photoreceptors cannot be regenerated from mammalian eyes [8]. Lately, specific cells that can be transplanted into the subretinal space have been found to integrate with host retina and restore some retinal function [9]. Cell replacement has been deemed as the most practicable and promising method of treating RD. Extracellular vesicles (EVs) are membrane-bound cellular products (30~1000 nm) that covering various types of soluble and trans-membrane proteins as well as nuclei acids, which contain microvesicles (MVs) and exosomes (EXOs) [10]. To date, EVs possess capacity to affect tumor regeneration, invasion, regeneration, and degenerative processes in addition to immune modulation, under physiological and pathological conditions [11]. Since Evans and Kauffman firstly derived pluripotent cell culture in 1981, human embryonic stem cells (hESCs) have long been identified as a potential cell provenance for a variety of diseases triggered by tissue dysfunction or loss [12]. Given its accessibility, stem cells have been used to generate photoreceptors that can be engrafted into diseased eyes [13]. However, how human embryonic stem extracellular vesicles (hESEVs) participate in RD progression remains unclear.

HSP90 is diffusely distributed in all retinal layers, from the tips of the outer segment to RPE cells, the retinal ganglion cells, and the inner segment [14]. As a ubiquitously expressed molecular chaperone, HSP90 is imperative for the post-translational stability of its client proteins, most of which are essential for cell differentiation, survival and growth [15]. In this research, we revealed that hESEVs could promote Müller cells to dedifferentiate and retrodifferentiate into retinal progenitor cells. The HSP90 in hESEVs could alleviate RD by Oct4 upregulation in Müller cells.

## Materials and methods

### Ethical statement

The experimental scheme was authorized by the Committee of Experimental Animals of Tianjin Medical University Eye Hospital. All procedures were in compliance with the Guide for the Care and Use of Laboratory Animals.

### Culture of hESCs

hESCs were purchased from the UCLA Broad Stem Cell Research Center Core facility. In feeder layer-free and serum-free conditions, hESCs were amplified in filtered mTeSR1 medium (Stem Cell Technologies, Vancouver, Canada). Then, hESCs were plated on culture flasks coated with 1.2% Matrigel (BD Biosciences, San Jose, CA), followed by incubation in a humidified incubator of 5% CO_2_ at 37 °C. Then, hESEVs were isolated and maintained with culture medium being refreshed every 24 h. hESCs were passaged once every 5~6 days by using dispase (Stem Cell Technologies) to keep colonies at 80% confluence. The microscopy was applied to observe signs of differentiation in hESC colonies.

### Isolation of hESEVs

After ultracentrifugation at 120,000×*g* with fetal bovine serum (FBS) (Gibco, NY, USA) for 18 h, exo-depleted FBS was obtained. Forty-eight hours before EXO isolation, the mTeSR1 medium was replaced with exo-depleted FBS. EXOs were harvested by differential centrifugation: the supernatant of culture medium was collected followed by low-speed centrifugation (300*g* × 10 min, 2000*g* × 10 min) at 4 °C, ultracentrifugation (10,000*g* × 70 min), and ultracentrifugation (100,000*g* × 70 min). hESEV precipitates were resuspended with appropriate phosphate buffer saline (PBS) and then subjected to ultracentrifugation (100,000*g* × 70 min) and resuspension with PBS for further measurement or packaged at − 80 °C avoiding repeated freezing and thawing.

### Identification of hESEVs, EXOs, and MVs

#### Electron microscope observation

The protein concentration of EXOs was measured utilizing a BCA kit (Beyotime, Shanghai, China) and adjusted to 200 μg/mL with PBS. Following standing at room temperature for 30 min, 5 μL of hESEVs, MVs, or EXOs were negatively stained with 2% (w/v) sodium phosphotungstate solution (Sigma-Aldrich, Merck KGaA, Darmstadt, Germany) at room temperature for 10 min. Then, these solution samples were dried naturally in air prior to observation and photograph under a transmission electron microscope (TEM, HT7700, Hitachi, Japan) (Scare bar = 100 nm).

#### Western blot

The 100 mL of hESEVs, MVs, or EXOs were lysed with RIPA lysis buffer on ice for 30 min and centrifuged at 120,000 r/min at 4 °C to obtain the supernatant. The supernatant (20 μL) was subjected to protein concentration measurement by a BCA method, and the rest supernatant was treated with 5 × SDS protein loading buffer in a ratio of 4:1. Then, the protein was boiled at 100 °C for 10 min and separated at the density of 20 μg/well by SDS-PAGE. The following steps were consistent with the western blot method.

### Nanosight tracking analysis

hESEVs, MVs, and EXOs were diluted in 1:10, and 1 mL of EXO solution was added in a quartz colorimetric utensil. Then, the particle size was determined by a dynamic light-scattering instrument (Nano S90, Marvin, UK).

### Establishment of RD rat model

Royal college of surgery (RCS) rats (*n* = 42) were grouped into RCS group (*n* = 18), PBS group (*n* = 12), and hESEVs group (*n* = 12). Rats in RCS group were divided into six groups: postnatal day 7 (P7), P15, P30, P60, P90, and P120 (*n* = 3). P30 rats in the hESEVs group were injected with 5 μL sterile PBS (sPBS) supplemented with hESEVs into the vitreous cavity by using a 33-g Hamilton syringe (Hamilton Company, Beltsville, MD). A 5 μL volume of sPBS was injected into rats in the PBS group. Then, rats in the PBS group and hESEVs group were separately divided into P30, P60, P90, and P120 (*n* = 3). Same-age Long-Evan’s rats (*n* = 21, purchased from Experimental Animal Center of the Field Research Institute of Daping Hospital of the Third Military Medical University) were used as the control group.

### Distribution of PKH67 labeled-hESEVs in vivo

To inspect the distribution and location of hESEVs in vivo, hESEVs were stained with the green fluorescent dye PKH67 (Sigma-Aldrich, Merck KGaA, Darmstadt, Germany). The specific procedure was as follows: EVs were concentrated to 200 μL using a 100 KD evaporator tube, followed by being suspended in 1 mL of diluent C to obtain solution A. Solution B was prepared after mixture of 4 μL of PKH67 dye and 1 mL of diluent C. Then, solution A was mixed with solution B for 2~5 min of staining. The staining was terminated by adding 2 mL of 1% BSA (since the dyeing was completed instantaneously, there was no need for long time incubation). The mixture was ultracentrifuged at 4 °C (100,000*g* × 6 h) to harvest the precipitate. Then, the precipitate was resuspended in 300 μL of PBS to obtain PKH67 dye-labeled hESEVs for subsequent experiments.

### Hematoxylin and eosin (H&E) staining

Rats were anesthetized and euthanized with 3% pentobarbital sodium (50 mg/kg, intraperitoneal injection) to obtain the eyeballs. Then, anterior segment of eyeballs was removed under a microscope. The ocular rings were fixed in 4% formaldehyde solution at 4 °C for 1 day and dehydrated with the gradient ethanol of 10%, 20%, and 30%. Then, the optic cups were embedded with optimal cutting temperature compound (OCT compound) followed by being frozen and sliced at 15 μm. The slides were subjected to polylysine prior to H&E staining.

### Immunofluorescence labeling in rats

Following PBS washing for 3 × 5 min, the retinal sections were incubated with 0.1% Triton for 10 min, subjected to normal rabbit serum (Invitrogen, CA, USA) to block non-specific binding sites, and then incubated at 37 °C for 30 min to discard serum. Then, primary antibody against Vimentin (NB300-223, 1:5000) or CHX10 (NBP1-84476, 1:1000) (Novus, St. Louis, MO, USA) was incubated with sections overnight in a humidified box at 4 °C (the NC group was treated with 0.01 mmol/L of PBS). Then, sections were washed thrice with PBS for 5 min, followed by incubation with the secondary antibody labeled with FITC fluorescence (ab6662, 1:1000, Abcam, USA) or Texas Red (ab6787, 1:1000, Abcam, USA) in a humidified box at 37 °C for 40 min. Following 5 min of PBS rinsing thrice, sections were sealed with water-soluble resin. Pictures were captured with a fluorescence microscope (Olympus IX71, Tokyo, Japan). Analyses of images were performed by using Fiji [16] and associated plugins as previously described [17].

### Cell transfection

hESCs in the logarithmic phase were transfected with 2 μg of pcDNA3.1a, pcDNA3.1a-HSP90, sh-NC, or sh-HSP90 by utilizing the Lipofectamine 2000 kit (Invitrogen, USA). Cells were immersed in serum-free Dulbecco’s modified Eagle medium (DMEM) for incubation in a constant temperature incubator at 37 °C gassed with 5% CO_2_. When cell growth was in good condition and the density reached 10^6^~10^7^, the culture solution was discarded. Then, cells were washed twice with PBS and cultured with DEME supplemented with 10% EVs-depleted FBS. The culture solution was changed every 24 h, and the supernatant was collected for storing at − 80 °C or for the extraction of hESEVs and isolation of MVs. MVs were named as pcDNA3.1a-MVs, pcDNA3.1a-HSP90-MVs, sh-NC-MVs, and sh-HSP90-MVs.

### Isolation of EXOs and MVs

Following differential centrifugation, hESEVs were divided into EXOs and MVs. The supernatant was harvested by centrifugation of hESC culture medium at 300*g*. Then, the supernatant was centrifuged at 2800*g* for 40 min to separate EXOs and MVs, followed by 60 min of ultracentrifugation at 16,500*g* at 4 °C to pellet MVs and 120 min of ultracentrifugation at 120,000*g* at 4 °C to pellet EXOs.

### Müller cell culture and grouping

The eyeballs of rats in P8~P10 were placed in DMEM at 4 °C for 6~8 h avoiding light and then transferred into digestive juice containing 0.1% trypsin, 0.02% EDTA, and 70 U/mL collagenase for 1 h of incubation at 37 °C. The digestion was then terminated, the anterior segment was removed, and the retina was isolated while avoiding contamination of the RPE and ciliary epithelium. The retina was mechanically dissociated into the polymer, followed by culture in DMEM containing 10% FBS. About 7 days later, the retinal polymer and debris were cleaned up and cells attached to the bottom of culture dish were maintained. Then, 5 days later, the cells were digested with trypsin and grown in DMEM supplemented with 10% FBS to further purify Müller cells. Following 12 h of incubation, Müller cells were treated with 1 mL PKH67 labeled-hESEVs, MVs, EXOs, sh-NC-MVs, sh-HSP90-MVs, pcDNA3.1-MVs, or pcDNA3.1-HSP90-MVs. Then, Müller cells were accordingly grouped into the hESEVs group, MVs group, EXOs group, sh-NC-MVs group, sh-HSP90-MVs group, pcDNA3.1-MVs group, and pcDNA3.1-HSP90-MVs group. A control group was set for comparison in which Müller cells were treated with DEME containing 10% EVs-depleted FBS. Müller cells were treated for 8, 12, 24, or 48 h for subsequent experiments.

### Labeling of hESEVs, MVs, and EXOs and uptake detection in Müller cells

The 100 μL suspension of hESEVs, MVs, or EXOs received 1 μL DiI (Santa Cruz Biotechnology, USA) for 1 h of incubation at 37 °C water bath avoiding light. Then, hESEVs, MVs, or EXOs were harvest by ultracentrifugation, followed by resuspension in PBS and − 80 °C storage. Müller cells (10^5^) were seeded into six-well plate and then cultured overnight in an incubator at 37 °C gassed with 5% CO_2_. After cells adhered to the wall, appropriate DiI labeled-hESEVs, MVs, or EXOs were added to the pretreated six-well plate for 24 h of incubation. Müller cells were stained with DAPI, and the uptake of EXOs was observed under a fluorescence microscope (BX51, Olympus, Tokyo, Japan).

### Cell immunofluorescence

To detect the expressions of specific markers in Müller cells (Vimentin) and retinal progenitors (CHX10), Müller cells were cultured in six-well plates which covered with coverslips for 24 h and subsequently starved in serum-free DMEM for 8 h. Following treatment with PKH67 labeled-hESEVs, MVs, EXOs, sh-NC-MVs, sh-HSP90-MVs, pcDNA3.1-MVs, or pcDNA3.1-HSP90-MVs, the Müller cells were washed thrice with PBS, followed by 20 min of fixation in 4% formaldehyde solution (Thermo Fisher Scientific, MA, USA) at room temperature and 10 min of permeation with PBS containing 0.1% triton-X-100. Then, cells were washed with PBS and blocked with sheep serum (Gibco, NY, USA) (1:50) for 30 min. Following 5 min of PBS washing thrice, cells were subjected to the primary antibody against Vimentin (NB300-223, 1:5000) or CHX10 (NBP1-84476, 1:1000) (Novus, St. Louis, MO, USA) overnight at 4 °C for incubation. Cells were washed with PBS thrice and incubated with Texas Red-labeled sheep anti-mouse IgG (ab6787, 1:1000, Abcam, USA) at room temperature for 2 h. The nuclei of Müller cells were stained with DAPI (4′,6′-diamidino-2-phenylindole; Vector Laboratories, Inc., Burlingame, CA) at room temperature for 5 min prior to capture the fluorescence images under immunofluorescence microscopy (Olympus IX71, Tokyo, Japan).

### Rat electroretinogram (ERG)

Rats were fed in dark-adapted conditions for 12 h with available ad libitum food and water before the experiment. Then, rats were anesthetized by intraperitoneal injection of 3% sodium pentobarbital (50 mg/kg). Tetracaine was applied to both eyes for local anesthesia and tropicamide for dilatation of the pupil. The pretreated rats were placed on a self-made experimental table. When testing one eye, the untested eye was covered with a black eye mask. Then, contact lens electrode was placed at the cornea as the recording electrode, while needle electrode placed on the ipsilateral cheek was used as reference electrode and needle electrode on the tail was used as ground electrode. The latent time and amplitude of the Rob-ERG b wave were recorded by rod cell detection system.

### Quantitative reverse transcription polymerase chain reaction (qRT-PCR)

The hESCs and Müller cells were dissolved in 1 mL of TRIzol (Thermo Fisher Scientific, MA, USA), and total RNA was extracted according to the specification. After quantification, the PCR reaction system was configured according to the PCR quantitative reagent kit (Takara, Dalian, China). The cDNA template was synthesized by reverse transcription reaction in a PCR amplification instrument, and the qRT-PCR experiment was carried out with ABI7500 real-time PCR system (Applied Biosystems, USA). The reaction conditions were 95 °C initial denaturation for 10 min, followed by 40 cycles of 95 °C denaturation for 10 s, 60 °C annealing for 20 s and 72 °C extension for 34 s. Then, the miRNA expression levels of HSP90 and Oct4 were analyzed. The internal reference was regarded as GAPDH, and data analysis employed the 2^−ΔΔCt^ method [18]. The formula is as follows: ΔΔCt = [Ct_(target gene)_ − Ct_(reference gene)_]_experimental group_ − [Ct_(target gene)_ − Ct_(reference gene)_] _control group_. All primers were synthesized by Genewiz Biotechnology Co., Ltd., and the amplified primer sequences of each gene and its primers are shown in Table 1.

**Table 1:** Primer sequence information

Name of primer	Sequences (5′-3′)
HSP90-F	CCTCAAGTCCACGCATCCAG
HSP90-R	TCGCCTCCTTGTGCATCTTC
Oct4-F	CAGGGTTTTTGGGATTAAGTTCT
Oct4-R	TGTGTCCCAGGCTTCTTTATTTA
GAPDH-F	ACCACAGTCCATGCCATCAC
GAPDH-R	TCCACCACCCTGTTGCTGTA

*F* forward primer, *R* reversed primer

### Co-immunoprecipitation (Co-IP)

The supernatant of protein was collected after Müller cells were transfected with sh-NC, sh-HSP90, pcDNA3.1a, or pcDNA3.1a-HSP90 for 48 h. Four hundred microliters of supernatant was coincubated with 10 μL of HSP90 antibody (ab13492, Abcam, USA) or IgG antibody (NC) overnight at 4 °C. The Protein G Agrose was added to the supernatant for 3~5 h of rotation at 4 °C. The mixed solution was centrifuged at 1000*g* for 5 min with the supernatant removed. The collected immunoprecipitate was washed in washing buffer (50 mM Tris-HCL/pH 7.4, 100 Mm NaCL, 5 mM CaCL_2_, 5 mM MgCL_2_, and 0.1% Nonidet P-40) for three times, and then precipitates were resuspended in 1 × SDS-PAGE loading buffer. The protein was subjected to a mental bath for 5 min at 100 °C and separated by electrophoresis with 10% PAGE prior to PVDF membrane transfer. The primary antibody against Oct4 (2750S, 1:1000, Cell Signaling Technology, MA, USA) and secondary antibody against goat anti-rabbit IgG (1:5000, ComWin Biotech Co., Ltd., Beijing, China) were used for western blot.

### Western blot

Following 48 h of cell transfection, Müller cells were washed thrice with precooled PBS buffer followed by being lysed with 100 μL/50 mL RIPA lysis buffer and placed on ice for 30 min. The Müller cells were centrifuged at 12,000 rpm at 4 °C for 10 min to acquire the supernatant, and then the supernatant was moved in a 0.5 mL centrifuge tube to preserve at − 20 °C or quantification by using a BCA kit (Vazyme, Nanjing, China). Then, the protein was treated with 6 × SDS loading buffer for denaturation at 100 °C, followed by 10% SDS-PAGE electrophoresis and 90 min of PVDF membrane transfer by using 4 °C precooled transfer buffer. After blocked in 5% nonfat dry milk-TBST for 1 h, the membranes were incubated with primary antibodies against TBST-CD9 (ab92726, 1:500), CD63 (ab59479, 1:500), CD81 (ab79559, 1:1000), TSG101 (ab83, 1:1000), Calnexin (ab112995, 1:1000, Abcam, USA), HSP90 (4875S, 1:1000), Oct4 (2750S, 1:1000), and β-actin (ab4970s, 1:1000) (Cell Signaling Technology, MA, USA) overnight at 4 °C. After washed three times with TBST for 10 min, the membranes were incubated with goat anti-mouse IgG or goat anti-rabbit IgG (1:5000, Beijing ComWin Biotech Co., Ltd., Beijing, China) for 2 h. The protein expression level was detected after TBST washing.

### Statistical analysis

Data were analyzed by utilizing SPSS 18.0 (IBM Corp., Armonk, NY, USA) and GraphPad Prism 6.0 (GraphPad Software Inc.). Data were presented as mean ± standard deviation (SD). The *T* test was applied to the comparison between two groups, and one-way analysis of variance (ANOVA) was used for comparison among multiple groups. The Bonferroni test was performed for post hoc test. A *P* < 0.05 was considered statistically significant.

## Results

### Retrodifferentiation of Müller cells during RD in RCS rats

RCS rat belongs to a rat with gene mutation of genetic receptor tyrosine kinase that leads to phagocytosis of rod outer segments (ROS) of photoreceptor cells by RPE, consequently resulting in photoreceptor cell death. Therefore, RCS rat is an ideal animal model of RD [19].

**Fig. 1:**
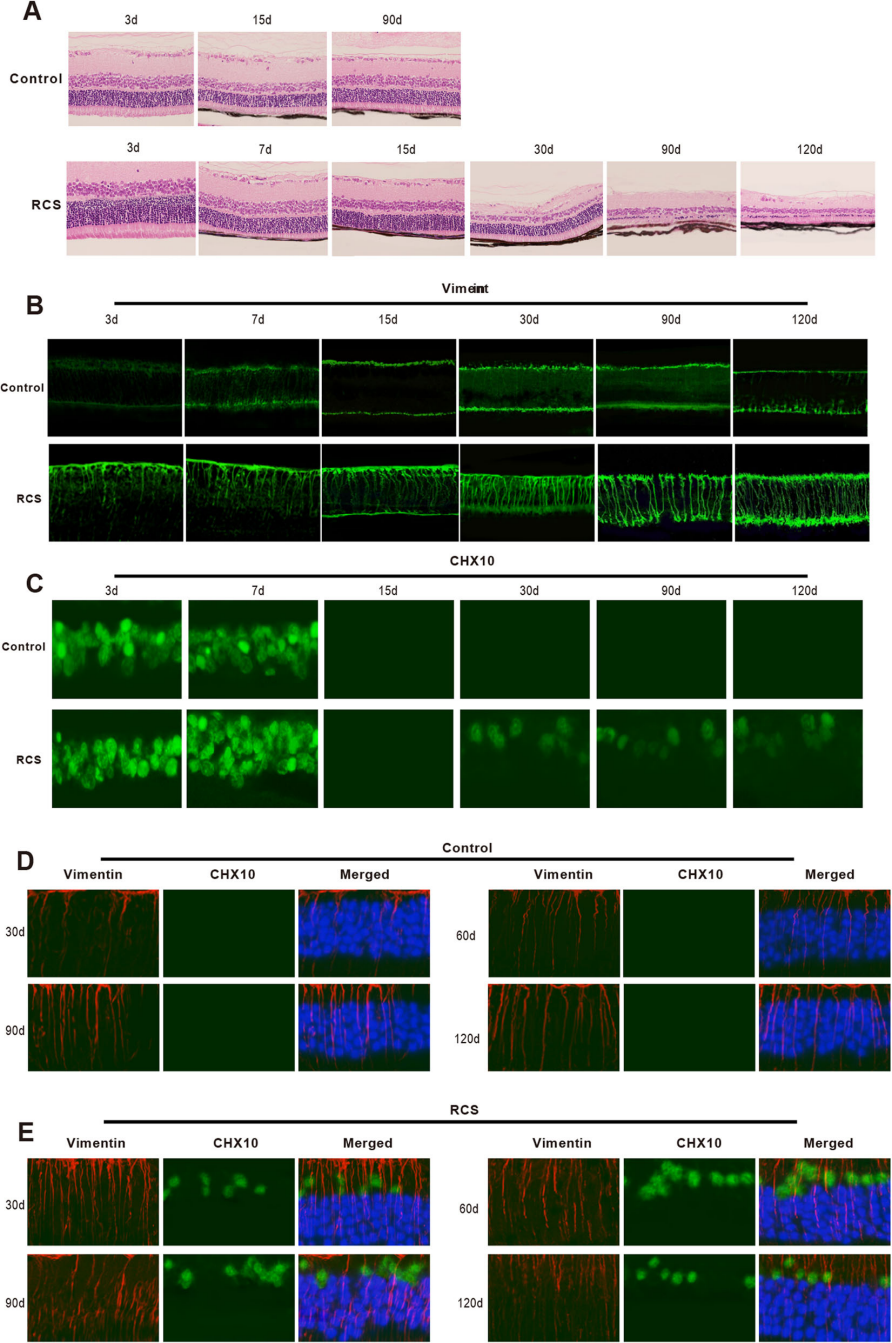
Müller cells retrodifferentiate into retinal progenitor cells in RD of RCS rat. In the RD process of RCS rat, H&E staining was used for the inspection of retinal damage (**a**), immunofluorescence for the detection of fluorescence intensities of Vimentin and CHX10 (**b**, **c**), and double immunofluorescence staining assay for the observation of Vimentin and CHX10 coexpression in the RCS group and control group (**d**, **e**); RD, retinal degeneration; RCS, royal college of surgery

H&E staining results of rats in the RCS group presented that there were disordered photoreceptor outer segments in rats at P15, fragmentation of the photoreceptor outer segments at P30, and stromal hyperplasia of photoreceptor outer segments, the disappearance of retinal outer nuclear layer as well as thinning of the inner nuclear layer at P90 and P120, whereas staining on rats in the control group showed that the structure of the retina is basically normal at P15 and P90 rats (Fig. 1a). These findings indicated that the retinal morphological changes of RCS rats began at P15 (eyes opened time), reached its peak at P30, and progressed to the late stage of RD at P90 and P120.

Immunofluorescence was performed to test the fluorescence intensity of Müller cell marker Vimentin. Müller cells of rats in the RCS group formed obvious long strip protuberances that spanned the entire depth of the neural retina at P15. The arrangement of these protuberances in the retinal nerve fiber layer was found to be disordered at P60, and Müller cells of rats became hypertrophic at P90. At P120, these protuberances extended to subretinal space to form the fibrous layer structure. Müller cells of rats in the control group appeared obvious long strip protuberances that arranged in order at P30 and at a later time (Fig. 1b).

After the detection of retinal progenitor cell marker CHX10, we found that in the RCS group, CHX10-positive cells were widely presented in the retina of rats at P7, disappeared at P15, and then reappeared dispersedly in the retinal inner layer at P30 to P120. However, CHX10-positive cells in the control group only extensively existed in the retina before opening eyes (Fig. 1c). These findings manifested that retinal progenitor cells in RCS rat widely existed in the retina since birth, disappeared after eye-opening (P15), reappeared at the peak of RD (P30), and persisted in advanced RD (P120).

Subsequently, the location of Vimentin and CHX10 was observed by double immunofluorescence staining. The results showed that a small number of Vimentin and CHX10 double-labeled positive cells were found in the retinal inner nuclear layer of rats at P30~P120 of the RCS group, and the cells were in round or oblong. Furthermore, all the CHX10-positive cells were double-labeled Vimentin. However, the control group had no CHX10-positive cells after Vimentin and CHX10 labeling immunofluorescence (Fig. 1d). These findings displayed that the retrodifferentiation of Müller cells in the RCS rats resulted in the re-emergence of retinal progenitor cells in the retina, whereas the control group did not undergo Müller cell retrodifferentiation.

Collectively, the above results indicated that the reappeared retinal progenitor cells in RCS rat at the peak of RD were triggered by the retrodifferentiation of retinal Müller cells. Additionally, retinal progenitor cells were gradually decreased after P30. Hence, in this study, RCS rats were selected to inject hESEVs at P30.

### hESEVs promote the retrodifferentiation of Müller cells to alleviate RD in RCS rats

**Fig. 2:**
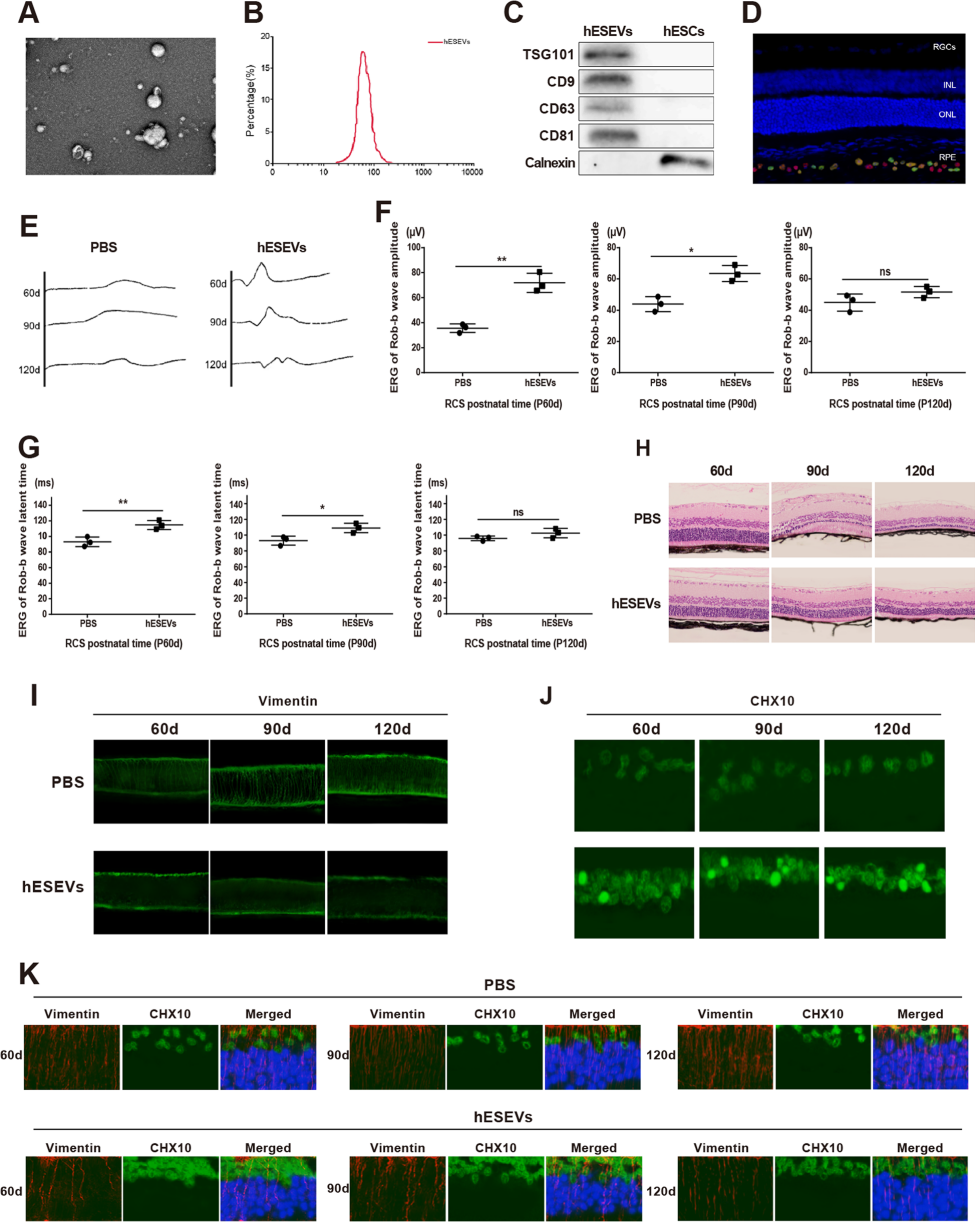
hESEVs alleviate RD of RCS rats by promoting Müller cell retrodifferentiation. TEM was employed for observation of hESEV morphology (**a**), NanoS90 for measurement of EVs size (**b**), and western blot for detection on the expressions of EV markers (**c**). Then, EV tracking method was used to observe EV distribution in retinal tissues, and we found that EVs were infiltrated into RPE cells (**d**). The retinal function (**e**), amplitudes, and latent times of Rod-b wave (**f**, **g**) in RCS rats of hESEVs group and PBS group were investigated by ERG. Subsequently, the damage of retina was evaluated by H&E staining (**h**), the fluorescence intensities of Müller cell marker (**i**), and progenitor cell marker (**j**) were analyzed by immunofluorescence, and coexpressions of Müller cell marker and progenitor cell marker were determined by double immunofluorescence staining (**k**); *P < 0.05, **P < 0.01 vs. the PBS group; TEM, transmission microscope; hESEVs, human embryonic stem cells; EVs, extracellular vesicles; RPE, retinal pigment epithelium; ERG, electroretinogram; PBS, phosphate buffer saline

hESEVs were purified by ultracentrifugation and differential centrifugation. A large number of round or elliptical vesicles can be seen under a TEM, with a complete membranous structure on the vesicle periphery and a bilayer membrane structure on partial vesicle periphery (Fig. 2a). qNano was employed to analyze the size of hESEV particles, and the results showed that the range of size distribution was 40~160 nm (Fig. 2b), which was consistent with the morphological characteristics of EVs. The specific proteins of hESEVs were detected by western blot. The results manifested that TSG101, CD63, CD9, and CD81 were expressed in isolated and purified hESEVs rather than in hESCs. Calnexin is a specific calcium-binding protein in the endoplasmic reticulum membrane that acts as a marker of cells and rarely presents in EVs. Subsequently, western blot results presented that Calnexin expression was observed in hESCs and no Calnexin expression was found in hESEVs (Fig. 2c), indicating EVs were successfully extracted.

To explore the effect of hESEVs on RD, PKH67-hESEVs were injected into the vitreous cavity of P30 RCS rats. EVs were noticed in RPE cells by EVs tracking method (Fig. 2d), demonstrating that EVs could be successfully injected into the retinal cells through the vitreous cavity.

ERG is a widely recognized electrophysiological index for evaluation of retinal function. The ERG results showed that the amplitudes of Rod-b wave (Fig. 2e, f, *P* < 0.05) and Max-b wave (Fig. 2e, *P* < 0.05) of rats at P60 and P90 were significantly higher in the hESEVs group than those of the PBS group, and the latent times of Rod-b (Fig. 2e, Fig. 2g, *P* < 0.05) wave and Max-b wave (Fig. 2e, f, *P* < 0.05) of rats at P60 and P90 were markedly lower in the hESEVs group than those of the PBS group. However, no obvious difference was observed in the hESEVs group and the PBS group with regard to the amplitude and latent time of the Rod-b wave and Max-b wave of rats at P120 (Fig. 2e–g). The above findings illustrated that hESEVs could protect retina.

Subsequently, H&E staining findings manifested that there were disorder and fragmentation of the photoreceptor outer segments in rats at P60 of the PBS group, and stromal hyperplasia of photoreceptor outer segments, disappearance of retinal outer nuclear layer as well as thinning of the inner nuclear layer at P90 and P120, whereas in the hESEVs group, photoreceptor cells arranged in an orderly manner in rats at P60, and photoreceptor outer segments appeared a few stromal hyperplasias accompanied by the visibility of retinal outer nuclear layer at P90 and P120 (Fig. 2h). These findings illustrated that hESEVs could alleviate retinal damage and RD.

Immunofluorescence results displayed that Müller cells of rats in the PBS group formed protuberances which arranged disorderly at P60, followed by hypertrophic at P90 and formation of the fibrous layer structure at P120. Müller cells in the hESEVs group formed obvious long strip protuberances that spanned the entire depth of the neural retina and arranged orderly (Fig. 2i). Meanwhile, there were few CHX10-positive cells in the PBS group, which diffused distribution in the retinal inner layer at P30 to P120. A large number of CHX10-positive cells were found in the retinal core layer of the hESEVs group which rats at P60 to P120 (Fig. 2j). The above results showed that hESEVs could promote the production of retinal progenitor cells in the process of RD of RCS rat.

Double immunofluorescence staining results showed that Vimentin and CHX10 double-labeled positive cells in the retinal inner nuclear layer of rats in the hESEVs group after P60 were more than those in the PBS group, and CHX10-positive cells were double-labeled Vimentin both in the PBS group and hESEVs group (Fig. 2k), indicating that hESEVs injected into the vitreous cavity of RCS rat with RD could promote the production of retinal progenitor cells which was originated from retinal Müller cell retrodifferentiation.

### hESEVs promote Müller cell retrodifferentiation in vitro

**Fig. 3:**
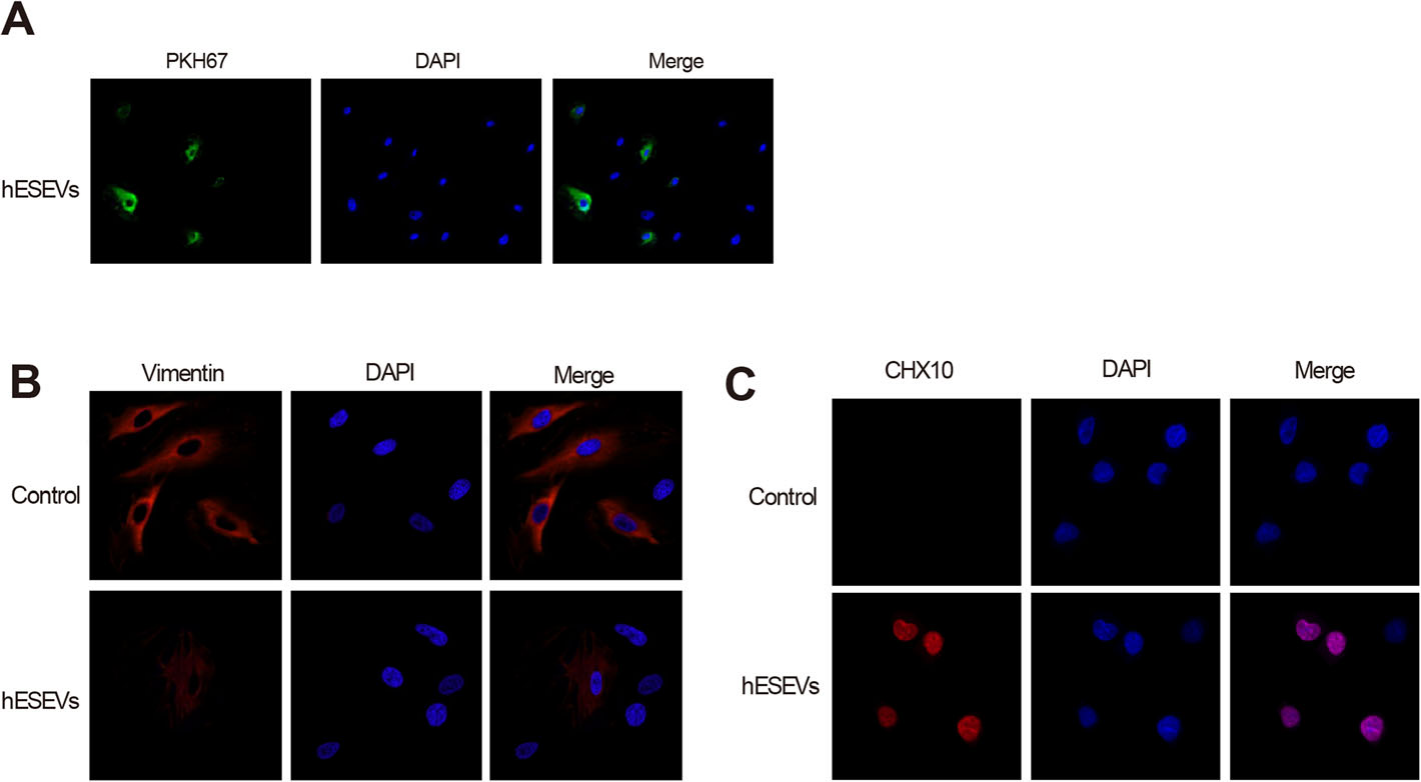
hESEVs promote the retrodifferentiation of Müller cells into retinal progenitor cells in vitro. Müller cells were treated with DiI labeled-hESEVs. Immunofluorescence was used to observe DiI labeled-hESEVs: green indicates DiI labeled-hESEVs and blue indicates DAPI-labeled nuclear DNA of Müller cells (bar = 25 μm) (**a**). The fluorescence intensities of Vimentin (**b**) and CHX10 (**c**) in retinal Müller cells at the 48th hours of treatment were assessed by immunofluorescence; hESEVs, human embryonic stem extracellular vesicles

To further explore whether hESEVs promotes Müller cell retrodifferentiation in vitro, Müller cells were exposed to DiI labeled-hESEVs. Green fluorescence was seen in Müller cells after immunofluorescence detection (Fig. 3a), revealing that hESEVs can be internalized by retinal Müller cells. Immunofluorescence detection of Vimentin was performed that fluorescence intensity was decreased in hESEVs group in comparison to the control group (Fig. 3b). The detection on CHX10 displayed that hESEVs group had enhanced fluorescence intensity than that in the control group, suggesting the increase of retinal progenitor cells (Fig. 3c). Taken together, all the above findings demonstrated that hESEVs could promote the dedifferentiation of Müller cells, and Müller cells may retrodifferentiate into retinal progenitor cells evidenced by strong fluorescence intensity of CHX10 after Müller cells treated with hESEVs.

### hESEVs promote Müller cell retrodifferentiation by MVs in vitro

**Fig. 4:**
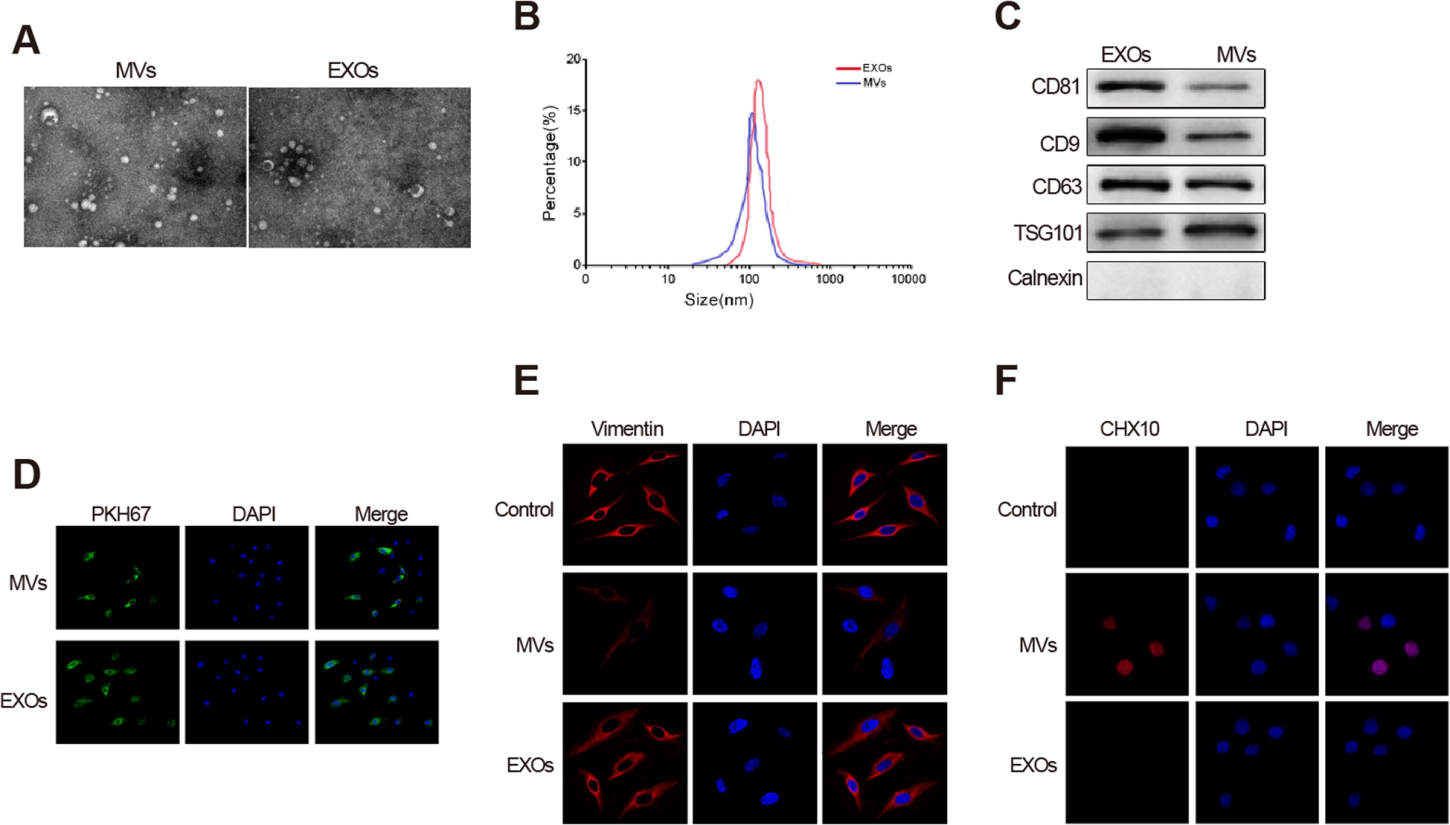
hESEVs boost retrodifferentiation of Müller cells through MVs in vitro. Müller cells were treated with DiI labeled-MVs or EXOs. Subsequently, TEM was utilized for measurement of morphologies of MVs and EXOs (**a**), NanoS90 for inspection of MV and EXO sizes (**b**), and western blot for determination of the expressions of MV and EXO specific proteins (**c**). DiI labeled-MVs or EXOs were observed by immunofluorescence: green indicates labeled-hESEVs and blue indicates DAPI labeled-nuclear DNA of Müller cells (bar = 25 μm) (**d**). The fluorescence intensities of Vimentin (**e**) and CHX10 (**f**) in retinal Müller cells were performed by immunofluorescence at the 48th hours of treatment; hESEVs, human embryonic stem extracellular vesicles; MVs, microvesicles; EXOs, exosomes; TEM, transmission microscope

To further investigate the mechanism of hESEVs on promoting the retrodifferentiation of retinal Müller cells, MVs and EXOs were isolated and extracted from hESEVs. The observation of TEM expounded that the diameter of MVs ranged from 90 nm to 1.1 μm and EXOs ranged from 20 to 120 nm. MVs are mainly distributed between 200 and 600 nm in diameter, and EXOs are mainly distributed in diameters between 40 and 80 nm (Fig. 4a, b). Western blot showed that the CD81, CD9, and CD63 proteins were highly expressed in EXOs rather than in MVs, and the protein expression of TSG101 was much higher in MVs than in EXOs (Fig. 4c). These findings indicated that MVs and EXOs were successfully isolated and extracted.

Subsequently, Müller cells were treated with DiI labeled-MVs and EXOs. The results manifested that there was increased green fluorescence in Müller cells of the MVs group and EXOs group, and the green fluorescence ultimately located in the cytoplasm of Müller cells (Fig. 4d). This indicated that MVs and EXOs could be internalized by retinal Müller cells. Immunofluorescence illustrated the increase in fluorescence intensity of CHX10 and a decrease in fluorescence intensity of Vimentin in the MVs group (Fig. 4e, f, vs. the control group). No obvious changes in fluorescence intensities of Vimentin and CHX10 were observed in the EXOs group and the control group. These data revealed that Müller cell was decreased and retinal progenitor cell number was increased in MVs. Taken together, these results revealed that hESEVs could promote Müller cells retrodifferentiated into retinal progenitor cells through MVs in vitro.

### MVs facilitate retrodifferentiation of Müller cells through HSP90

**Fig. 5:**
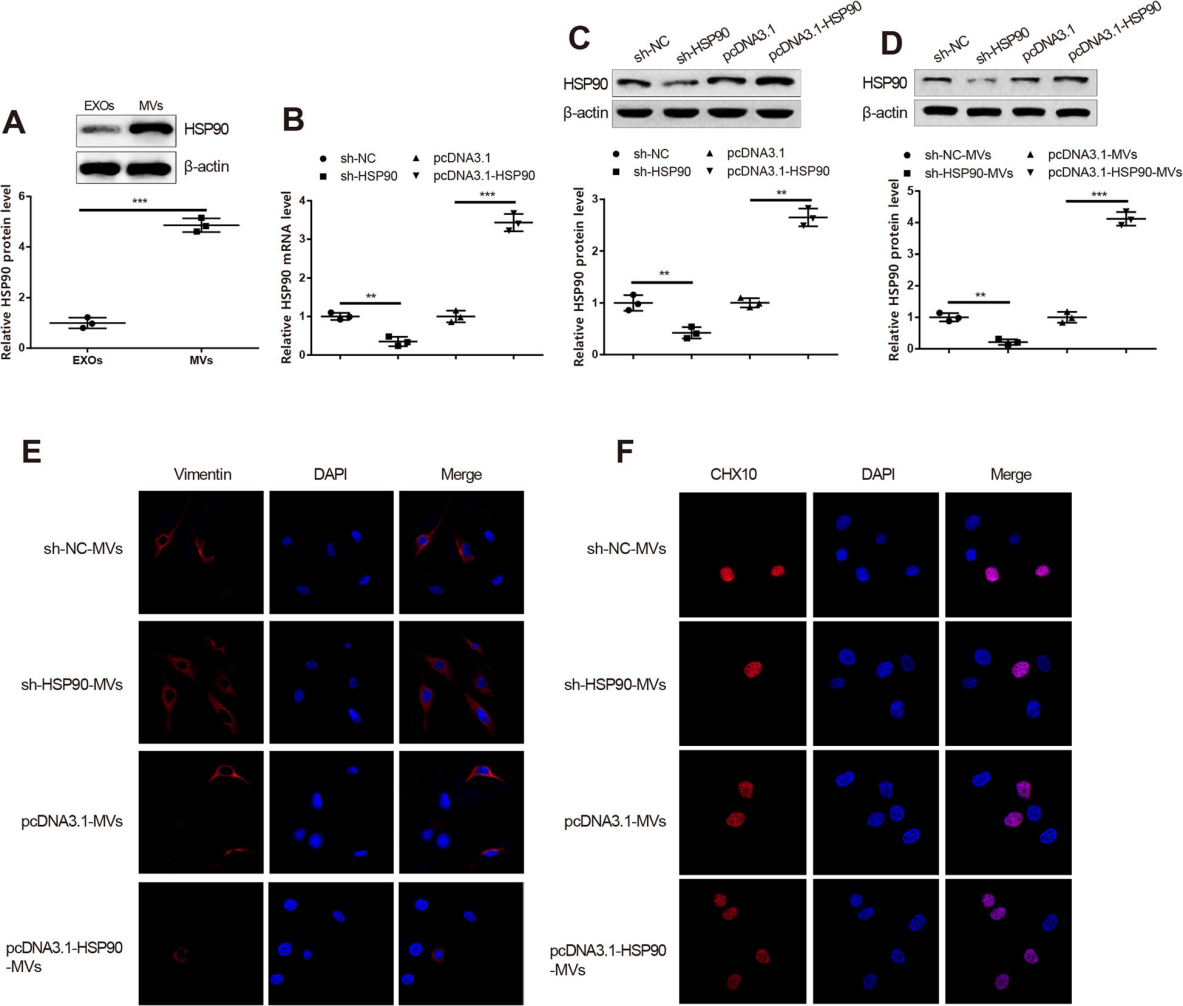
HSP90 in MVs enhances retrodifferentiation of Müller cells. Western blot analysis for HSP90 expression in MVs and EXOs (**a**); ***P < 0.001 vs. the EXOs group. qRT-PCR (**b**) and western blot (**c**) were employed for measurement of the mRNA and protein expressions of HSP90 in hESCs. After hESCs transfected with sh-NC, sh-HSP90, pcDNA3.1, or pcDNA3.1-HSP90, western blot was used to determine the protein expression of HSP90 in sh-NC-MVs, sh-HSP90-MVs, pcDNA3.1-MVs, or pcDNA3.1-HSP90-MVs groups (**d**); **P < 0.01, ***P < 0.001. Following 48 h of exposure to sh-NC-MVs,sh-HSP90-MVs, pcDNA3.1-MVs, or pcDNA3.1-HSP90-MVs, the fluorescence intensities of Vimentin (**e**) and CHX10 (**f**) in Müller cells were measured by immunofluorescence; MVs, microvesicles; EXOs, exosomes; hESCs, human embryonic stem cells; NC, negative control

The following study was arranged to probe the mechanism by which MVs boosted Müller cells to retrodifferentiate into retinal progenitor cells. The result performed that MVs had higher protein expression of HSP90 than EXOs (Fig. 5a). Then HSP90 was overexpressed or suppressed to verify whether MVs could contribute to retrodifferentiation of Müller cells through HSP90. Western blot presented that there was suppressed mRNA and protein expressions of HSP90 in the sh-HSP90 group rather than in the sh-NC group, and the pcDNA3.1-HSP90 group had markedly elevated HSP90 expression in comparison to the pcDNA3.1 group, manifesting good performance of HSP90 silencing and overexpression (Fig. 5b, c, *P* < 0.01). Subsequently, MVs were extracted and the result showed that there were heightened HSP90 expression levels in the sh-NC-MVs group (*P* < 0.01, vs. the sh-HSP90-MVs group) and the pcDNA3.1-HSP90-MVs group (*P* < 0.01, vs. the pcDNA3.1-MVs group) (Fig. 5d), indicating that knockdown or overexpression of HSP90 in retinal Müller cells could correspondingly alter the expression level of HSP90 in MVs.

After 48 h of exposure to PKH67 labeled-sh-NC-MVs, sh-HSP90-MVs, pcDNA3.1-MVs, or pcDNA3.1-HSP90-MVs, Müller cells possessed higher expression of Vimentin in the sh-HSP90-MVs group (Fig. 5e, *P* < 0.01, vs. the sh-NC-MVs group) and elevated CHX10 expression in the pcDNA3.1-HSP90-MVs group (Fig. 5f, vs. the pcDNA3.1-MVs group), while Müller cells had a lower level of Vimentin in the pcDNA3.1-HSP90-MVs group (Fig. 5e, vs. the pcDNA3.1-MVs group) and suppressed CHX10 expression in the sh-HSP90-MVs group (Fig. 5f, vs. the sh-NC-MVs group), illustrating that the promotive role of MVs in Müller cell retrodifferentiation could be refrained by knockdown of HSP90 in MVs. The above findings were consistent with the biological function of HSP90 that HSP90 is a molecular chaperone involved in cell development, growth, and differentiation.

### HSP90 in MVs mediates Oct4 expression in Müller cells and interacts with Oct4

**Fig. 6:**
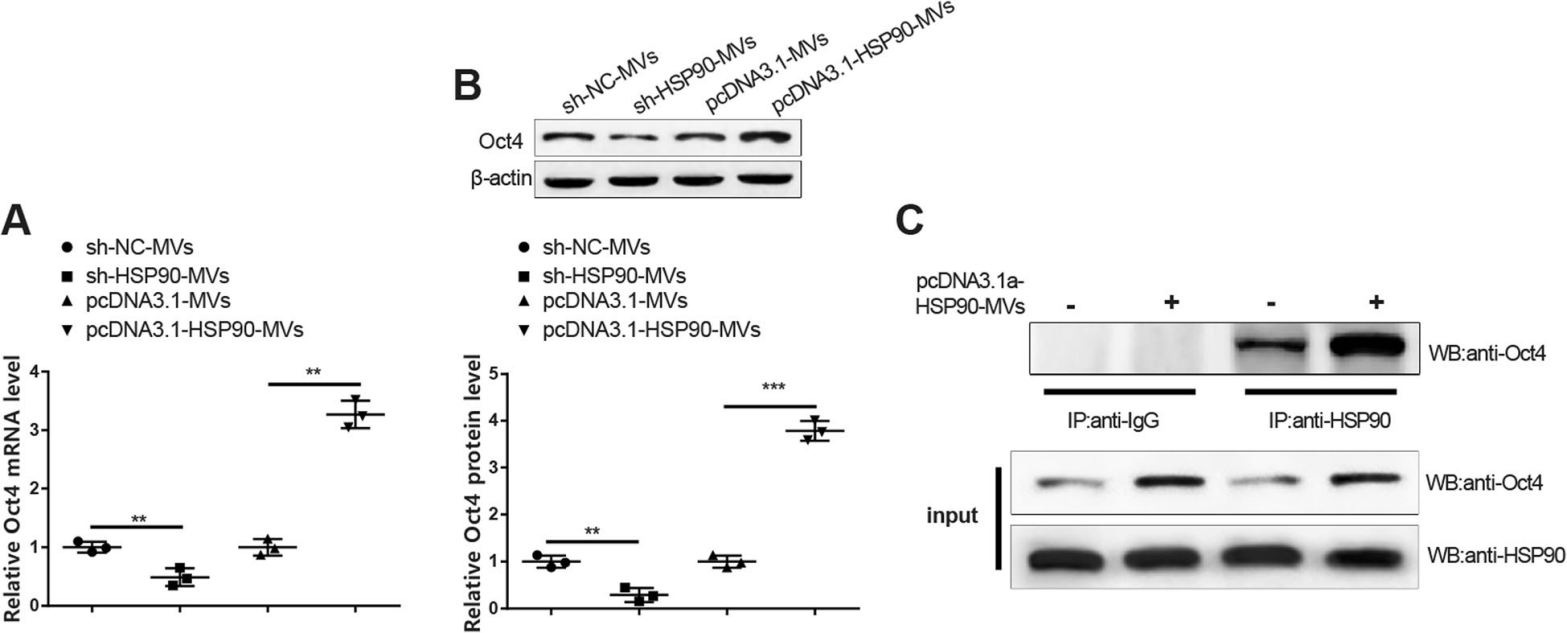
HSP90 in MVs regulates and interacts with Oct4 in Müller cells. After Müller cells were transfected with sh-NC-MVs, sh-HSP90-MVs, pcDNA3.1-MVs, or pcDNA3.1-HSP90-MVs for 48 h, qRT-PCR (**a**) and western blot (**b**) were used for analysis of the mRNA and protein expressions of Oct4, and Co-IP for measurement of the interaction between HSP90 and Oct4 after HSP90 was internalized by Müller cells (**c**); **P < 0.01, ***P < 0.001; MVs, microvesicles

In recent years, HSP90 is responsible for the occurrence and progression of the tumor by serving as a target for many oncoproteins. Hence, Müller cells were treated with sh-NC-MVs, sh-HSP90-MVs, pcDNA3.1-MVs, or pcDNA3.1-HSP90-MVs for 48 h to verify whether HSP90 in MVs promotes Müller cell retrodifferentiation by mediating protein expression in Müller cells. Western blot obtained that there were increased mRNA and protein expressions of Oct4 in the pcDNA3.1-HSP90-MVs group (Fig. 6a, b, *P* < 0.01, vs. the pcDNA3.1-MVs group), and decreased Oct4 expression in sh-HSP90-MVs group (Fig. 6a, b, *P* < 0.01, vs. the sh-NC-MVs group), presenting that HSP90 in MVs facilitates Oct4 expression in Müller cells.

Co-IP was carried out to ascertain whether HSP90 acts as a molecular chaperone to interact with Oct4 and stabilize Oct4 expression. Observation from Co-IP demonstrated that Oct4 brand can be detected when antibody of HSP90 was used for immunoprecipitation, while no Oct4 brand was found when antibody of IgG was used for immunoprecipitation. Expressions of both Oct4 and HSP90 were detected in total cell lysate (Fig. 6c). These results revealed the interaction of HSP60 and Oct4. Furthermore, Oct4 is reported to facilitate Müller cell retrodifferentiation [20]. Collectively, MVs could facilitate Müller cells to retrodifferentiate into retinal progenitor cells by regulation of Oct4 expression in Müller cells by HSP90.

## Discussion

Diseases that result in loss of retinal cells, such as RD, often motive the permanent visual impairment because the retina, like other central nervous system tissues, has poor regenerative capacity in humans [21]. Repairing of the retina with replacement cells derived from hESCs may provide the groundwork for new means to treat RD [22]. In this study, Müller cells were exposed to hESEVs to test the potential functions and possible mechanisms of hESEVs in RD. Our findings demonstrated that HSP90 in hESEVs could mitigate RD by facilitating retinal Müller cell retrodifferentiation by upregulating Oct4 expression.

As the principal glia of the retina, Müller cells span the entire thickness of the retina, support the surrounding neurons, including photoreceptors, and are engaged at the formation and maintenance of the blood-retinal barrier [23]. In addition, the uniquely positioned Müller cells exhibit diverse functions to maintain retinal homeostasis [24]. For example, Jenny R. Lenkowski et al. have verified that in teleost fish, Müller glia can generate and regenerate retinal neurons [25]. Thus, Müller cells are imperative targets for inspections of retinal regeneration. In consistent with the previous study, we further manifested that Müller cells could retrodifferentiate into retinal progenitor cells in RCS rat evidenced by high fluorescence intensity of CHX10 and low Vimentin expression existed in RCS rat under RD. hESEVs are an influential therapeutic biologics for in vivo delivery which is extensively applied for the regulation of immune responses, promotion of endogenous progenitor proliferation, remodeling of extracellular matrices, and stimulation of angiogenesis [10]. EVs, agents to transfer genetic information, are the basis of biological courses and elicit therapeutic capacity in tissue regeneration in kinds of organ degenerative diseases such as the liver [26], kidney [27], lung [28], and heart [29]. In this study, we assessed the expression profiles of EVs from hESCs in Müller cells by immunofluorescence. Collective evidence revealed that EVs from hESCs could promote Müller cell retrodifferentiation. Subsequently, we characterized hESEVs and two of their fractionated subpopulations, MVs and EXOs. The effects of EXOs and MVs on Müller cells in vitro were distinguished and assessed by flow cytometry. Most interesting, we recognized that Müller cells could retrodifferentiate into retinal progenitor cells after treatment with EVs. However, the mechanism underlying the influence of MVs on RD progression has not been fully demonstrated.

The former study has revealed that prolonged HSP90 inhibition gives rise to photoreceptor cell death [30]. Additionally, HSP90 is found to confer a crucial effect on maintaining the stable connection between photoreceptor cells and Müller cells in the retina [31]. Therefore, HSP90 may play an indispensable role in the homeostasis of the retina. With regard to biological function of HSP90, we hypothesized that HSP90 had the capacity to mitigate RD. Western blot analysis corroborated the assumption described above that there was elevated protein expression of HSP90 in MVs. In addition, overexpression of HSP90 greatly contributed to the generation of retinal progenitor cells, whereas knockdown of HSP90 inhibited Müller cells dedifferentiated and retrodifferentiated into retinal progenitor cells. To probe the downstream protein of HSP90 in Müller cells, we screened Oct4 to seek further evidence for the potential synergistic effect of Oct4 on the regulation of RD. Western blot, qRT-PCR, and Co-IP displayed that HSP90 in MVs could upregulate Oct4 expression and interact with Oct4. As a POU-domain transcription factor, high expression of Oct4 has been discovered in maintaining cellular reprogramming and pluripotency in stem cells [32]. The previous study possesses similarities to our findings that HSP90 directly interacts with Oct4 and prevents it from degrading through the ubiquitin-proteasome pathway [33]. Taken together, HSP90 regulated and interacted with Oct4 to facilitate Müller cells to retrodifferentiate into retinal progenitor cells, thus alleviating RD.

## Conclusions

In brief, evidence in this study supported the promotive effect of hESEVs on the alleviation of RD. HSP90 in MVs could promote Müller cells dedifferentiate and retrodifferentiate into retinal progenitor cells by regulation of Oct4. Our findings here indicate that therapeutic strategies targeting MVs may be broadly applied to patients with RD and other neurodegenerative diseases and can be successful in prolonging the survival of endangered photoreceptors and in deferring irreversible vision loss. Nonetheless, these results may be limited by the risk of immune responses to the transplanted hESEVs in human patients.

## Supplementary Information

**Additional file 1.**

## Data Availability

The datasets used and/or analyzed during the current study are available from the corresponding author on reasonable request.

## Abbreviations

hESEVs: Human embryonic stem extracellular vesicles
hESCs: Human embryonic stem cells
MVs: Microvesicles
EXOs: Exosomes
RD: Retinal degeneration
RP: Retinitis pigmentosa
RPE: Retinal pigment epithelium
EVs: Extracellular vesicles
PBS: Phosphate buffer saline
RCS: Royal college of surgery
sPBS: Sterile phosphate buffer saline
P7/15/30/60/90/120d: Postnatal 7/15/30/60/90/120 day
H&E: Hematoxylin and eosin
OCT compound: Optimal cutting temperature compound
qRT-PCR: Quantitative reverse transcription polymerase chain reaction
Co-IP: Co-immunoprecipitation
ROS: Rod outer segments
ERG: Electroretinogram
