# Correction to: Human embryonic stem cell-derived extracellular vesicles alleviate retinal degeneration by upregulating Oct4 to promote retinal Müller cell retrodifferentiation via HSP90

**DOI:** 10.1186/s13287-021-02213-z

**Published:** 2021-02-15

**Authors:** Yifeng Ke, Xiaoe Fan, Rui Hao, Lijie Dong, Min Xue, Liangzhang Tan, Chunbo Yang, Xiaorong Li, Xinjun Ren

**Affiliations:** 1 Tianjin Key Laboratory of Retinal Functions and Diseases, Tianjin International Joint Research and Development Centre of Ophthalmology and Vision Science, Eye Institute and School of Optometry, Tianjin Medica lUniversity Eye Hospital, No 251, Fukang Road, Nankai District, Tianjin, 300384 People’s Republic of China; 2 Jincheng People’s Hospital, Jincheng, 048000 Shanxi People’s Republic of China; 3 Tianjin Eye Hospital, Tianjin Key Laboratory of Ophthalmology and Vision Science, Nankai University Eye Hospital, Clinical College of Ophthalmology, Tianjin Medical University, Tianjin, 300020 People’s Republic of China; 4 Department of Ophthalmology, Anhui No.2 Provincial People’s Hospital, Hefei, 230000 Anhui People’s Republic of China; 5 Institute of Genetic Medicine, Newcastle University, Central Parkway, Newcastle upon Tyne, NE1 3 BZ UK

**Correction to: Stem Cell Res Ther 12, 21 (2021)**

**https://doi.org/10.1186/s13287-020-02034-6**

**Correction**

The original article [1] contained an error in the Corresponding Authorship notation and Authors’ contributions sections which have since been corrected.

## References

[1] Ke Y (2021). Human embryonic stem cell-derived extracellular vesicles alleviate retinal degeneration by upregulating Oct4 to promote retinal Müller cell retrodifferentiation via HSP90. Stem Cell Res Ther.

