# Supplementary material for: Human embryonic stem cell-derived extracellular vesicles alleviate retinal degeneration by upregulating Oct4 to promote retinal Müller cell retrodifferentiation via HSP90
Source: Stem Cell Res Ther. 2021 Jan 7;12:21. doi: 10.1186/s13287-020-02034-6 (PMC7792097; doi:10.1186/s13287-020-02034-6)
Supplement: Supplementary file 1 — Additional file 1. [file 13287_2020_2034_MOESM1_ESM.docx]

**The first experiment:**


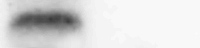


fig2C CD9


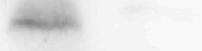


fig2C CD63


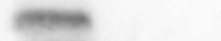


fig2C CD81


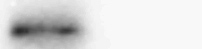


fig2C TSG101


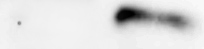


fig2C calnexin


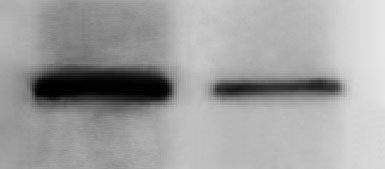


fig4C-CD9


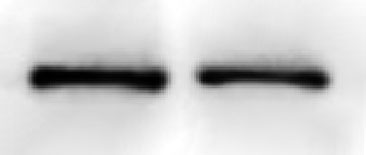


fig4C-CD63


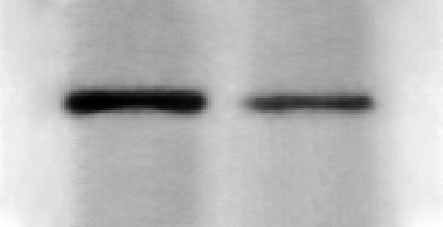


fig4C-CD81


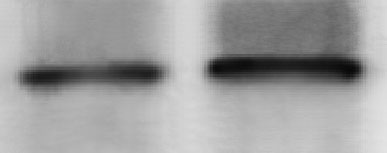


fig4C-TSG101


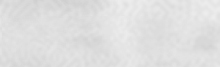


fig4C-Calnexin


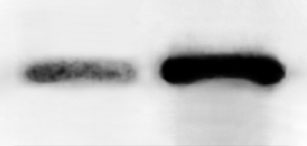


fig5A-HSP90


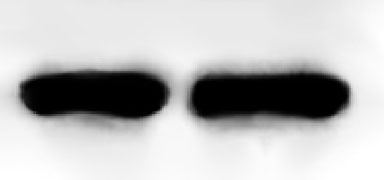


fig5A-β-actin


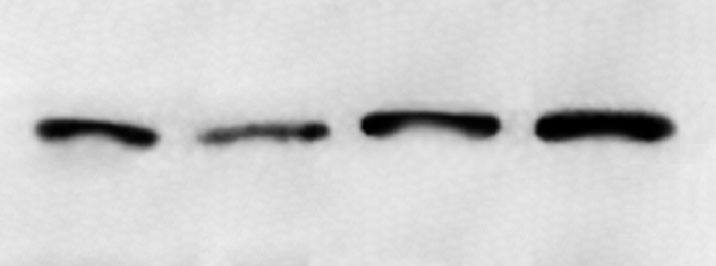


fig5C-HSP90


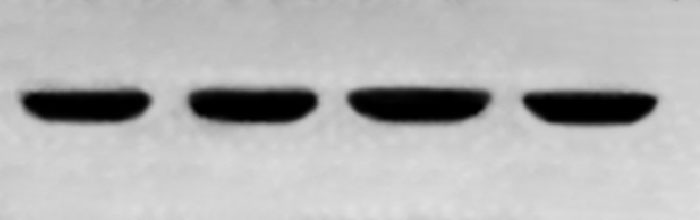


fig5C-β-actin


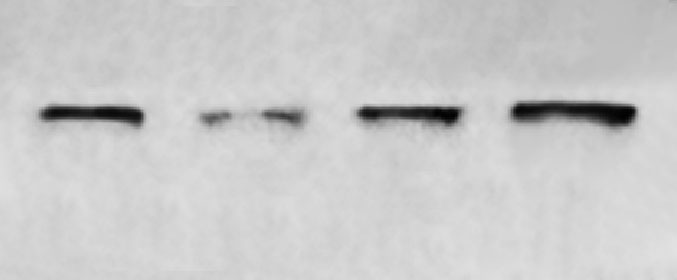


fig5D-HSP90


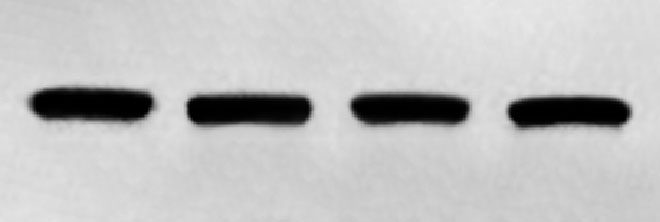


fig5D-β-actin


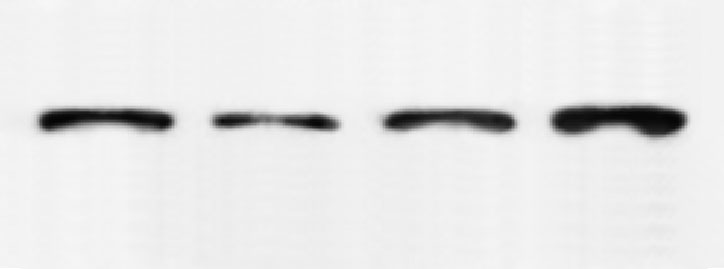


fig6B-Oct4


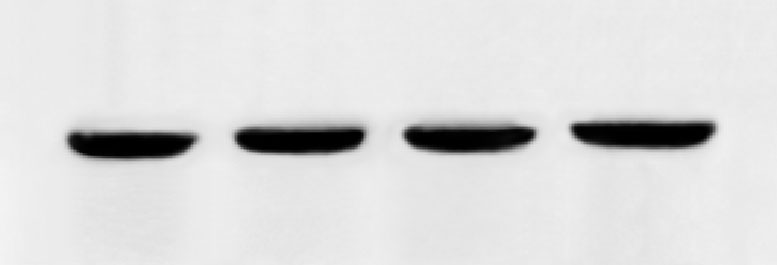


fig6B-β-actin


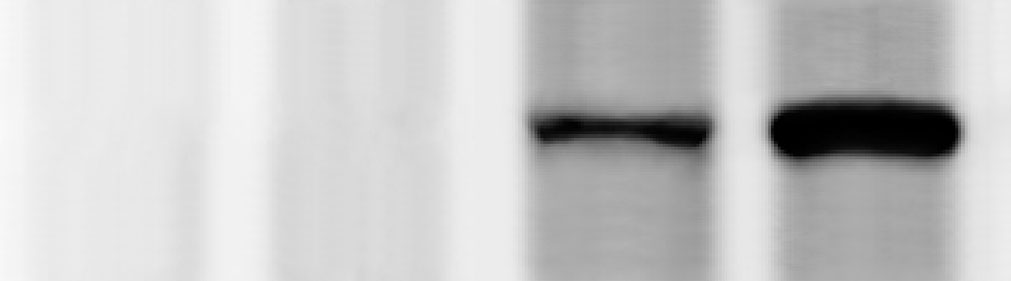


fig6C-IP-anti-Oct4


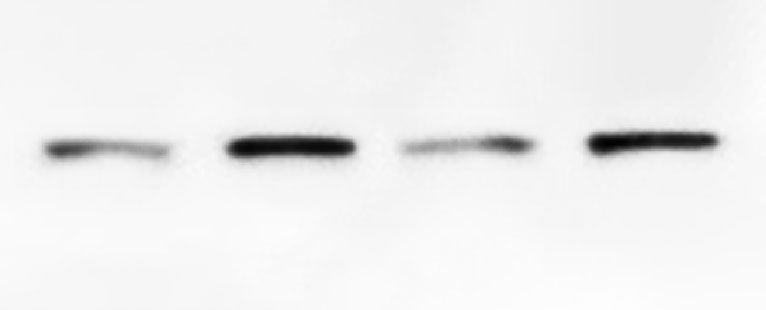


fig6C-input-anti-Oct4


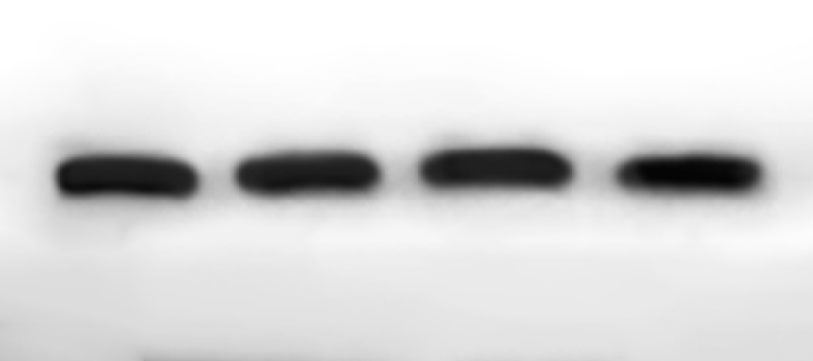


fig6C-input-anti-HSP90

**The second experiment**


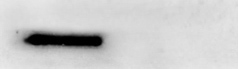


fig2C CD9


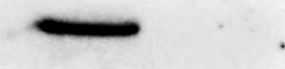


fig2C CD63


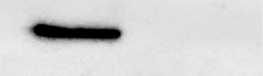


fig2C CD81


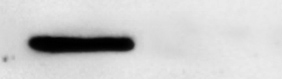


fig2C TSG101


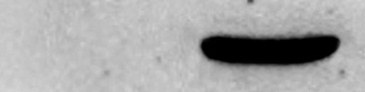


fig2C calnexin


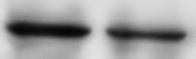


fig4C CD9


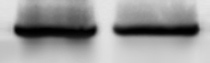


fig4C CD63


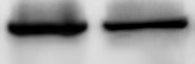


fig4C CD81


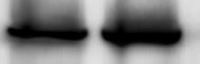


fig4C TSG101


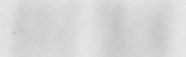


fig4C Calnexin


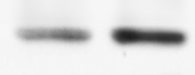


fig5A HSP90


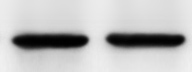


fig5A β-actin


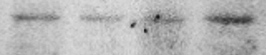


fig5C HSP90


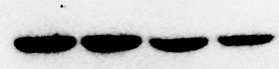


fig5C β-actin


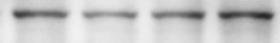


fig5D HSP90


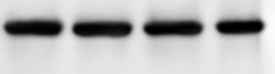


fig5D β-actin


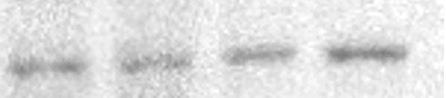


fig6B Oct4


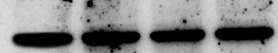


fig6B β-actin


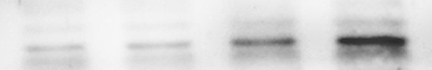


fig6C-IP-anti-Oct4


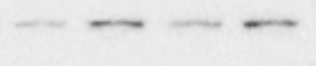


fig6C-input-anti-Oct4


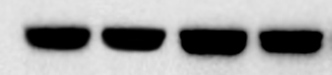


fig6C-input-anti-HSP90

**The third experiment:**


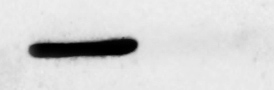


fig2C CD9


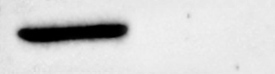


fig2C CD63


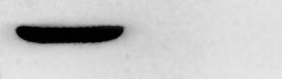


fig2C CD81


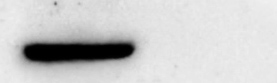


fig2C TSG101


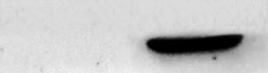


fig2C calnexin


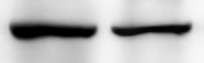


fig4C CD9-2


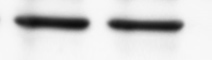


fig4C CD63


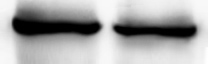


fig4C CD81


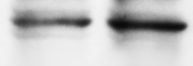


fig4C TSG101


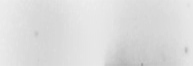


fig4C Calnexin


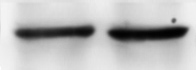


fig5A HSP90


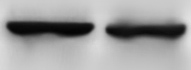


fig5A β-actin


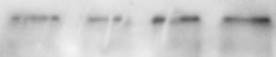


fig5C HSP90


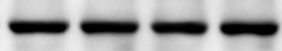


fig5C β-actin


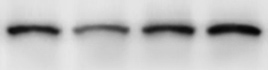


fig5D HSP90


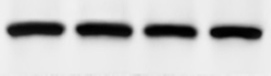


fig5D β-actin


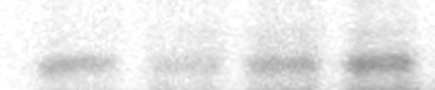


fig6B Oct4


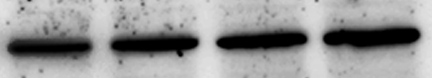


fig6B β-actin


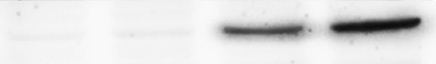


fig6C IP-anti-Oct4


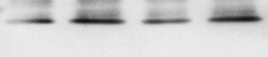


fig6C input-anti-Oct4


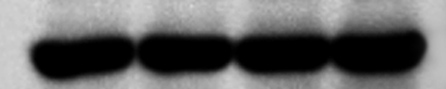


fig6C input-anti-HSP90

**The repeat result of FIG 1**


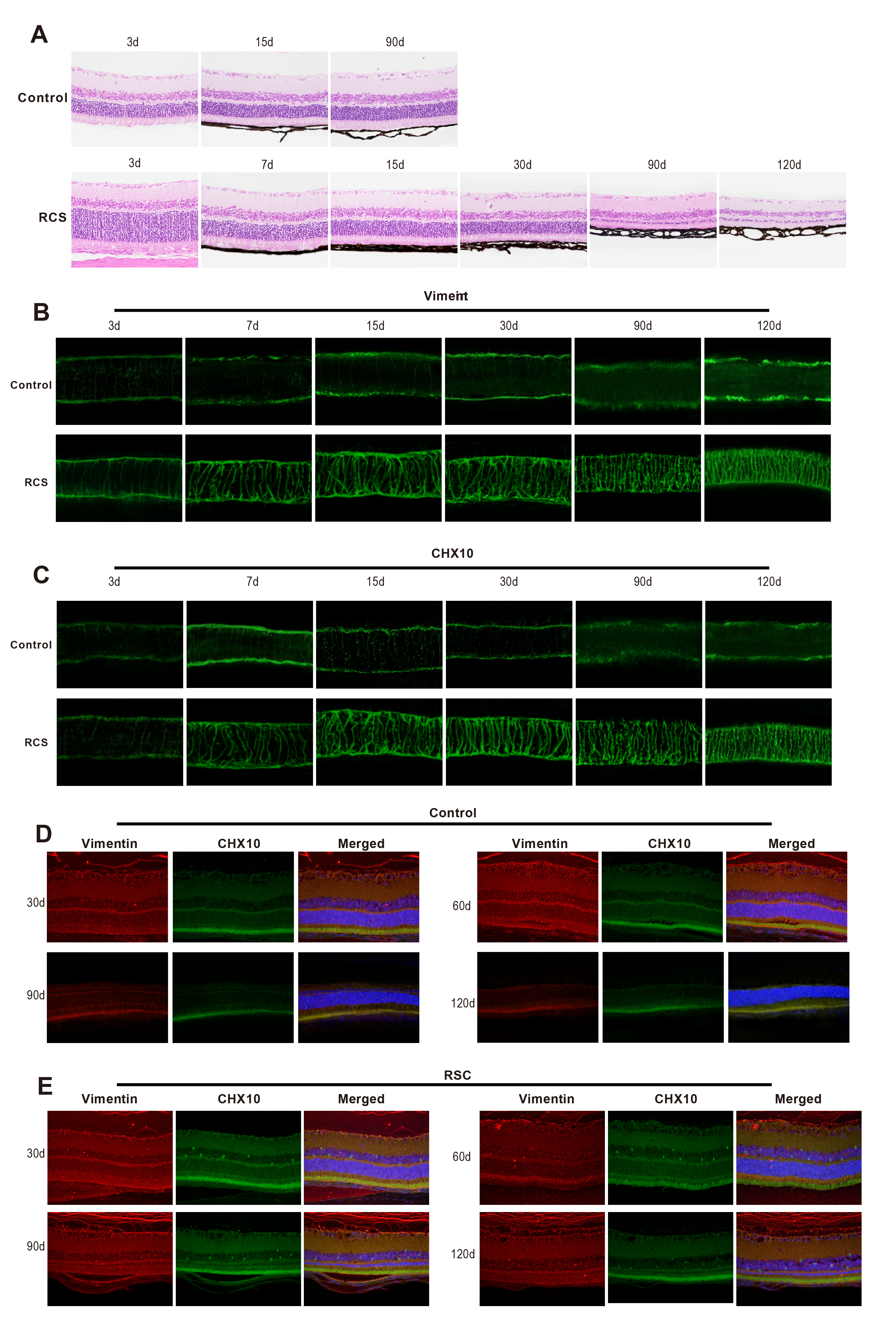


Fig 1 in second experiment


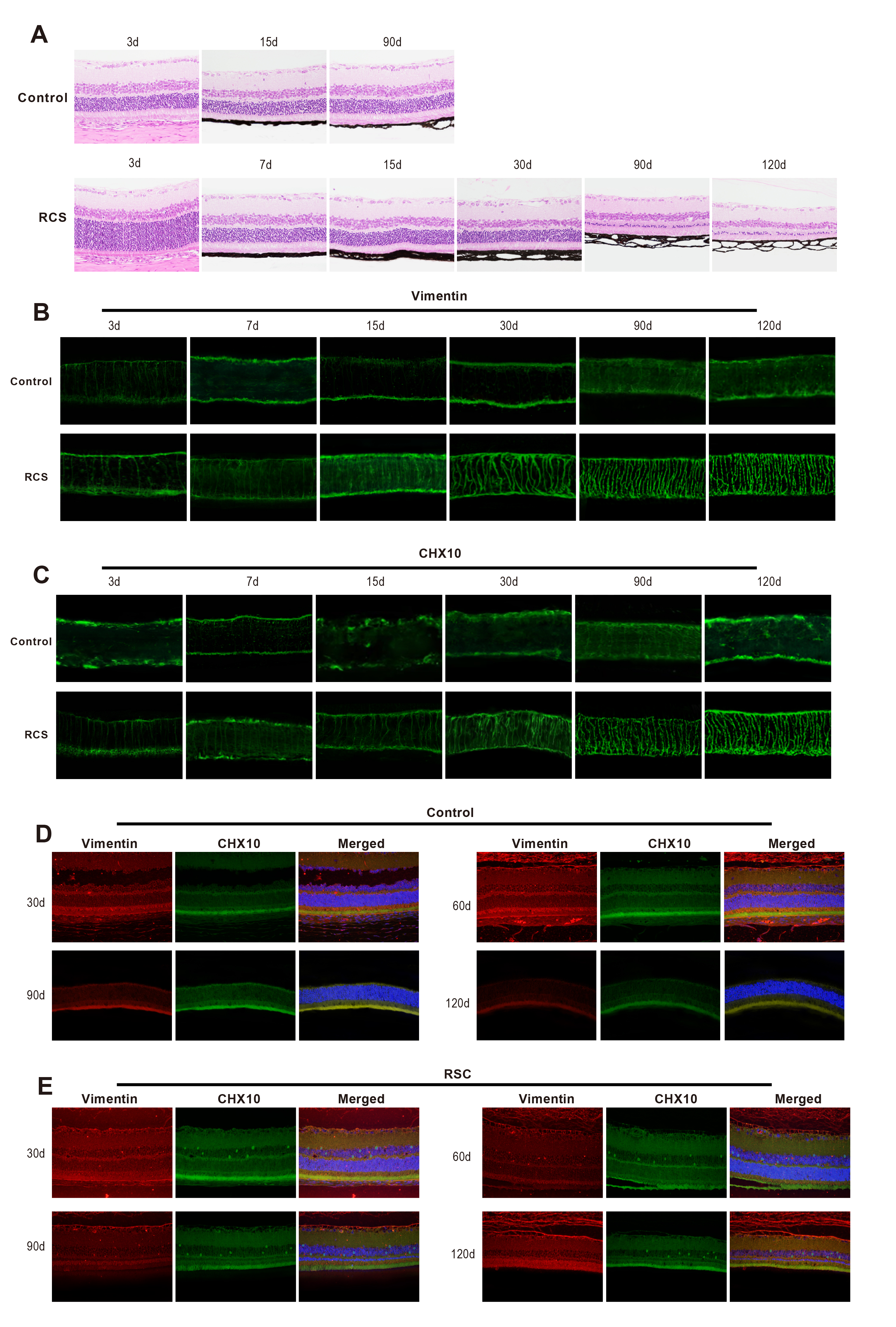


Fig 1 in third experiment
